# Unraveling and rectifying the free-riding behavior among students in university flipped classrooms: a uninorm DEMATEL method

**DOI:** 10.3389/fpsyg.2025.1617001

**Published:** 2025-06-09

**Authors:** Shiyu Yan, Lisheng Jiang, Zhili Zuo, Lixuan An, Li Wang

**Affiliations:** ^1^College of Management Science, Chengdu University of Technology, Chengdu, China; ^2^KERMIT, Department of Data Analysis and Mathematical Modelling, Ghent University, Ghent, Belgium; ^3^School of Architecture and Civil Engineering, Xihua University, Chengdu, China

**Keywords:** flipped classroom, free-riding, DEMATEL, uninorm operator, unfairness indices

## Abstract

**Introduction:**

Flipped classrooms move education toward a more student- and learning-centered pedagogy and practice. When flipped classrooms are applied, a free-riding phenomenon may occur when certain students in a group do not participate completely in group tasks but receive the same grade as the other students, which creates unfairness and is not conducive to the sustainable development of university education. As far as we know, quantifying the unfairness caused by free-riding in flipped classrooms is still a problem that needs to be addressed.

**Methods:**

This paper proposes a fair assessment framework to unravel and rectify student free-riding in flipped classrooms. Firstly, the uninorm DEcision-MAking Trial and Evaluation Laboratory (DEMATEL) method is proposed to generate comprehensive indices of students. Secondly, the unfairness indices of groups and the discount parameters of students are determined based on the comprehensive index. The discounted scores are employed to modify students' achievement to ensure fairness.

**Results:**

A case study involving 57 students is presented to demonstrate the applicability of the proposed method, and a sensitivity analysis is conducted to assess its robustness. Four findings are uncovered. (1) Free-riding behavior exists in university flipped classrooms, and quantifying unfairness enables more targeted pedagogical interventions. (2) Discounted scores can enhance student motivation while promoting fairness. (3) Group scores do not show a direct correlation with unfairness indices. (4) The number of students does not exhibit a direct correlation with unfairness indices.

**Discussion:**

The fair assessment framework provides educational administrators with a tool for quantifying the effectiveness of course implementation, promoting the positive development of collaborative learning and the effective implementation of flipped classrooms, and contributing to the sustainable development of university education.

## 1 Introduction

The ability to engage in active learning and collaborative learning is one of the core competencies that university students must possess (Canavesi and Ravarini, 2024; Padilla-Petry et al., 2025). As education changes, improving old teaching and learning methods to get better results has become very important. Good education reform needs to balance changes in teaching and learning, so that both teachers and students stay motivated, with the main goal of improving how well students learn (Ou and Chen, 2024). Many efforts around the world have been made in this area. Among them, the flipped classroom has become a key example of effective education reform (Fisher et al., 2024).

The flipped classroom has received much attention because it moves education toward a more student- and learning-centered pedagogy and practice. The flipped classroom model consists of three distinct phases: (1) pre-class preparation, (2) in-class learning activities, and (3) post-class consolidation (DeLozier and Rhodes, 2017). A common instructional approach in flipped classrooms involves assigning pre-class tasks to small groups of students (Sarwar et al., 2024). During pre-class preparation, student groups engage in planning and develop relevant materials for the assigned tasks, such as creating PowerPoint slides to support their presentations. During in-class learning activities, the groups present their work and collaboratively solve problems with their intra-group members. Meanwhile, the teacher poses guiding questions and assesses the quality of each group's presentation. Post-class consolidation is facilitated through after-school quizzes and homework assignments, which help reinforce the knowledge gained during the lessons. In such an interactive teaching system, a free-riding phenomenon (Meijer et al., 2020; Swaray, 2012) may arise, wherein some students fail to fully engage in group tasks but receive the same grade as other students. This situation creates an unfair environment for students who diligently contribute to group work. Identifying free-riding students is often challenging for teachers, as teachers typically do not directly participate in pre-class preparation activities.

Research on flipped classrooms can broadly be categorized into non-empirical and empirical studies. The former primarily involves theoretical and literature-based definitions and interpretations (Cheng et al., 2022), while the latter refers to research in which investigators independently collect data to formulate or test hypotheses (Shen, 2024). Empirical research employs methods such as quantitative approaches (e.g., questionnaires) (Awidi and Paynter, 2019), qualitative approaches (e.g., interviews) (Ma et al., 2024), or mixed-methods approaches (Han et al., 2024). In empirical research, the research objects of flipped classrooms can be divided into three categories: (1) Student feedback surveys (Caraballo Vidal et al., 2024). These focus on students' satisfaction with or opinions on online resources, flipped classroom teaching models, and learning environments (Ghafouri et al., 2024). (2) Teacher feedback surveys (Thai et al., 2024). These examine teachers' evaluations of flipped classroom teaching models and learning environments, as well as their instructional design, discourse strategies, and assessment literacy. (3) Combined teacher-student feedback surveys (Faro et al., 2024). These studies collect feedback from both teachers and students to provide a comprehensive understanding of flipped classroom practices (Etemi et al., 2024). In empirical studies, the results of flipped classroom research can be categorized into two groups: (1) to verify the effectiveness of flipped classrooms in improving the quality of education and teaching. Flipped classrooms have been shown to improve the satisfaction and achievements of students in many subjects, like medical education (Johnson et al., 2025), physical education (Ghorbel et al., 2025), and management education (Zhang et al., 2024; Rossouw and Steenkamp, 2025). (2) to find the factors that affect the effectiveness of flipped classrooms. Such as large language models (Teng et al., 2024), background variables (Hwang, 2024), pre-course materials (Chen, 2024a), and teaching interaction (Chen, 2024b), have been discovered to affect the effectiveness of flipped classrooms.

Empirical research has widely demonstrated the advantages of the flipped classroom, but most of them are from a macro perspective, and there is a lack of consideration of free-riding in practical application. To avoid the unfairness caused by free-riding, a straightforward idea is to ask students to evaluate each other (Weaver and Esposto, 2012). Through student evaluation, the influence of each student on other students can be analyzed, based on which the level and quality of student engagement in group tasks can be measured. To realize the above ideas, this paper establishes a fair assessment framework that takes into account intra-group student evaluation and teacher evaluation. The contributions and innovations of this paper are summarized as follows.

(1) Proposing the uninorm DEMATEL method. Firstly, the DEMATEL method (Sorooshian et al., 2023) is used to generate the centrality and causality indices of students, which reflect the influence of each student on other students. Then, the uninorm aggregation operator (Yager and Rybalov, 1996) is applied to integrate the centrality and causality indices, based on which a participation index is defined to characterize the level and quality of student engagement in group tasks.

(2) Defining the unfairness index and discounted scores. The participation indices are used to define the unfairness indices of groups and calculate the discounted scores of students to avoid the unfairness caused by free-riding.

(3) A case study involving 57 students and one teacher is given to show the potential of the uninorm DEMATEL method in addressing real-world problems. Sensitivity analysis is performed to demonstrate the robustness of the proposed method.

This paper is organized as follows: Section 2 reviews the uninorm aggregation operator and the DEMATEL method. Section 3 presents the fair assessment framework consisting of the uninorm DEMATEL method and the fair assessment index. Section 4 details a case study with sensitive analysis. Section 5 discusses the findings and implications of the case study. Section 6 summarizes the main findings of this paper, and Section 7 gives the limitations and future study.

## 2 Preliminaries

To facilitate the understanding of the model proposed in this paper, the DEMATEL method and the uninorm aggregation operator are reviewed in Sections 2.1, 2.2, respectively.

### 2.1 The DEMATEL method

The Decision-Making Trial and Evaluation Laboratory (DEMATEL) was initially developed by the Geneva Research Center of the Battelle Memorial Institute. To identify the correlation (causality) between alternatives, the DEMATEL method uses pairwise comparisons that capture the interrelationships between alternatives. A detailed review of the DEMATEL method can be found in Sorooshian et al. (2023). The procedure of the DEMATEL method is summarized as follows.

**Step 1. Obtain the initial matrix** **M**. For a pair of alternatives *a*_*i*_ and *a*_*j*_, a decision maker (e.g., a student in one group) is asked how much *a*_*i*_ influences *a*_*j*_. A scale of 0 to 100 can be used to make the recommendation, with 0 (resp. 100) representing no influence (resp. very high influence). The decision maker's response is denoted by $x_{j}^{i}$, representing the degree of the influence of *a*_*i*_ on *a*_*j*_. For the problem including *n* alternatives, the initial evaluation matrix has *n* rows and *n* columns, and its structure is displayed as


$$\begin{array}{l} M=\left[\begin{array}{cccc} x_{1}^{1} & x_{1}^{2} & \ldots & x_{1}^{n}\\ x_{2}^{1} & x_{2}^{2} & \ldots & x_{2}^{n}\\ \vdots & \vdots & \ddots & \vdots \\ x_{n}^{1} & x_{n}^{2} & \ldots & x_{n}^{n} \end{array}\right]\;. \end{array}$$

It is noted that $x_{j}^{i}=0$ (*i* = *j*; *i, j* = 1, 2, …, *n*).

**Step 2. Obtain the normalized evaluation matrix** $\bar{M}$. The normalized evaluation matrix is calculated by $\bar{M}=M/S$ where $S=\max\left\{\underset{i_{1}}{\max}\left\{\underset{i_{2}}{\sum }\overset{i_{2}}{\underset{i_{1}}{x}}\right\},\underset{i_{2}}{\max}\left\{\underset{i_{1}}{\sum }\overset{i_{2}}{\underset{i_{1}}{x}}\right\}\right\}$.

**Step 3. Calculate the total influence matrix** **T**. The direct total influence matrix is calculated by $T=\bar{M}{\left(I-\bar{M}\right)}^{-1}$ where *I* is an identity matrix. The total influence of *a*_*i*_1__ on *a*_*i*_2__ is represented by the element in column *i*_2_ of row *i*_1_ in the total influence matrix *T*, denoted by $t_{i_{1}}^{i_{2}}$.

**Step 4. Construct the influential relation map**. Based on *T*, the influence of *a*_*i*_ on the other alternatives is calculated by $e_{i}^{r}=\underset{i_{1}}{\sum }t_{i}^{i_{1}}$, which form the out influence vector $E_{r}={\left[e_{i}^{r}\right]}_{1\times n}$. The impact of the other alternatives on *a*_*i*_ is reflected by $e_{i}^{c}=\underset{i_{1}}{\sum }t_{i_{1}}^{i}$, which form the inner influence vector $E_{c}={\left[e_{i}^{c}\right]}_{1\times n}$. For instance, the total influence matrix is shown below.


$$\begin{array}{l} T=\left[\begin{array}{ccc} 2 & 3 & 3\\ 2 & 2 & 2\\ 2 & 3 & 2 \end{array}\right] \end{array}$$

The influence of *a*_1_ on the other alternatives is calculated by $e_{1}^{r}=\underset{i}{\sum }t_{1}^{i}=2+3+3=8$. The impact of the other alternatives on *a*_1_ is calculated by $e_{1}^{c}=\underset{i}{\sum }t_{i}^{1}=2+2+2=6$.

**Step 5. Calculate the centrality and causality indices**. The centrality index $\mu_{i}=e_{i}^{r}+e_{i}^{c}$ indicates the importance of alternative *a*_*i*_ to the entire decision problem. The causality index ${\text{λ}}_{i}=e_{i}^{r}-e_{i}^{c}$ shows the total net influence of *a*_*i*_ on the other alternatives. When λ_*i*_ > 0, *a*_*i*_ has an impact on other alternatives; when λ_*i*_ < 0, other alternatives affect *a*_*i*_. Continue to the above example, the centrality index of alternative *a*_1_ is calculated by $\mu_{1}=e_{1}^{r}+e_{1}^{c}=14$. The causality index of alternative *a*_1_ is calculated by ${\text{λ}}_{1}=e_{1}^{r}-e_{1}^{c}=2$.

In the field of education, the DEMATEL has been widely applied to (1) identify the factors that promote or hinder student learning (Chen et al., 2023; Ma et al., 2020; Lu et al., 2024), (2) find the factors that make students stressful (Garg and Bhardwaj, 2024), and (3) analyze learning design (Pourhejazy and Isaksen, 2024). In other fields, it has been employed in business process evaluation (Ni et al., 2025), operational risk factor assessment (Zheng et al., 2024), and key success factors recognition (Zhang et al., 2022). Given the good performance of the DEMATEL method in the field of education, this paper uses the DEMATEL method to assess the level and quality of student engagement in group tasks.

### 2.2 The uninorm aggregation operator

**Figure 1:**
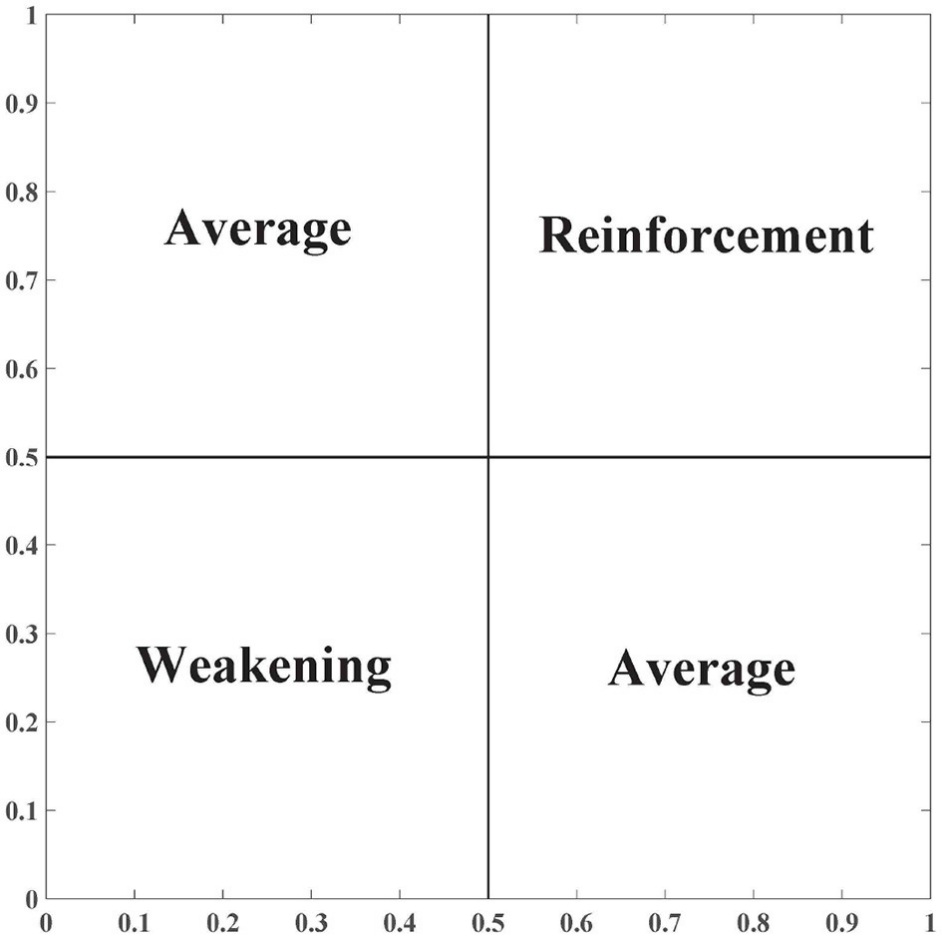
Four areas when *g* = 0.5.

As a generalization of t-norm and t-conorm, the uninorm aggregation operator (Yager and Rybalov, 1996; Fodor et al., 1997; De Baets, 1999) is a mapping *R*:[0, 1]^2^ → [0, 1]. A popular uninorm aggregation operation is (Wang et al., 2024)


(1) $$U(x_{1},x_{2})=\{\begin{array}{ll} x_{1}\times x_{2}÷g & ,\;if\;0\leq x_{1},x_{2}\leq g\\ \left(x_{1}+x_{2}-x_{1}\times x_{2}-g)÷(1-g\right) & ,\;if\;g\leq x_{1},x_{2}\leq 1\\ (x_{1}+x_{2})÷2 & ,\;else \end{array}$$

where *g* ∈ [0, 1] is the neutral element. The neutral element *g* divides the plane [0, 1]^2^ into four areas: one reinforcement area, one weakening area, and two average areas. In Figure 1, when the neutral element *g* rises, the reinforcement area is reduced while the weakening area is expanded.

Corresponding to the reinforcement and weakening areas, the uninorm aggregation operator has two features: reinforcement and weakening. An example is given to illustrate these two features.

** Example 1**. ***(Reinforcement and weakening of the uninorm aggregation operator)*** Consider two evaluations *x*_1_ and *x*_2_. The neutral element *g* is set to 0.5. When both *x*_1_ and *x*_2_ are larger than 0.5, *U*(*x*_1_, *x*_2_) is larger than *x*_1_ and *x*_2_, referred to as the reinforcement feature. For instance, let *x*_1_ = 0.6 and *x*_2_ = 0.7. Referring to Equation 1, *U*(*x*_1_, *x*_2_) = 0.76, which is larger than 0.6 and 0.7. When both *x*_1_ and *x*_2_ are smaller than 0.5, *U*(*x*_1_, *x*_2_) is smaller than *x*_1_ and *x*_2_, referred to as the weakening feature. For instance, let *x*_1_ = 0.3 and *x*_2_ = 0.2. Referring to Equation 1, *U*(*x*_1_, *x*_2_) = 0.12, which is smaller than 0.3 and 0.2.

Since its introduction, the uninorm aggregation operator has been widely applied in multiple criteria decision-making (Wang et al., 2024), group decision-making (Jin et al., 2024; Wu et al., 2022; Gong et al., 2023), and recommended systems (Palomares et al., 2018). Given that the uninorm aggregation operator performs well in aggregating the information given by decision makers, this paper uses the uninorm aggregation operator to integrate the evaluations of teachers and students.

## 3 Fair assessment framework

**Figure 2:**
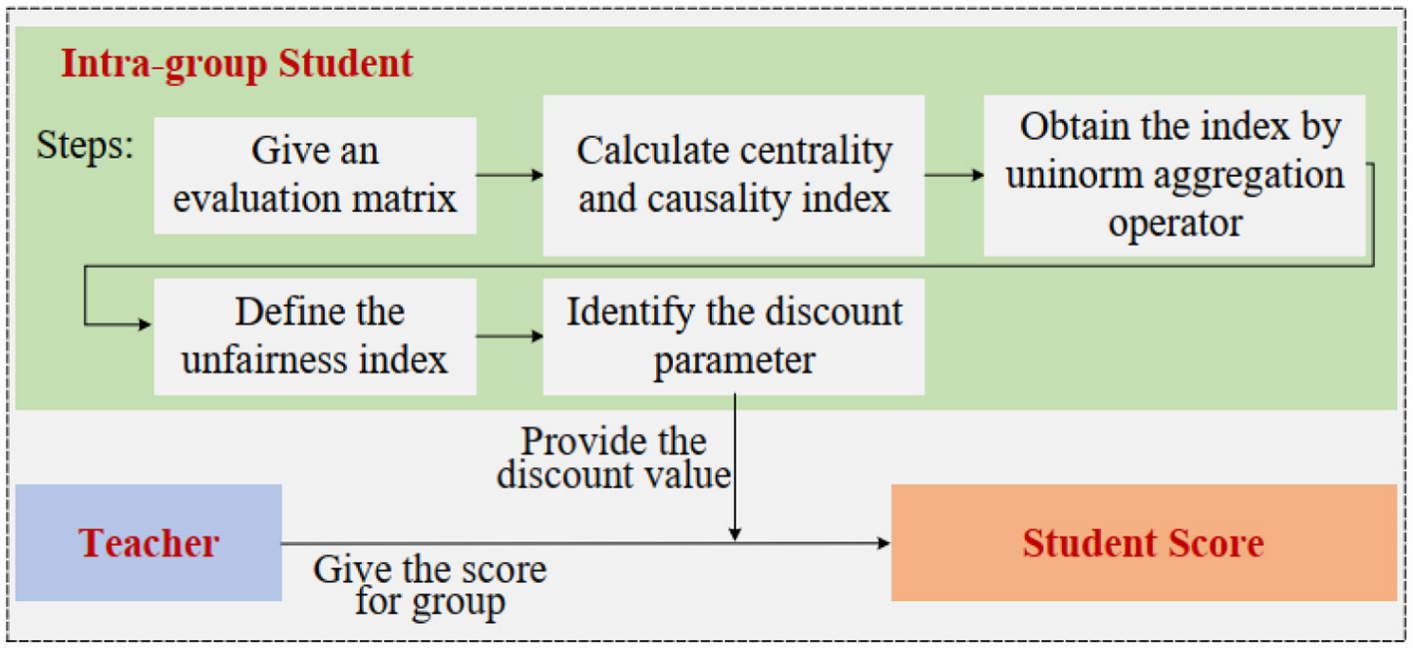
The framework of the evaluation study.

This section introduces a fair assessment framework, which consists of the uninorm DEMATEL method and the assessment indices, as illustrated in Figure 2. In Section 3.1, the uninorm DEMATEL method is constructed step-by-step. In Section 3.2, two indices are proposed to measure the unfairness of student groups and modify students' achievement to ensure fairness.

### 3.1 The uninorm DEMATEL method

In this section, the uninorm DEMATEL method is introduced to evaluate the level and quality of student engagement based on the evaluations given by students within the group.

Consider a group of *n* students represented as *A* = {*a*_*i*_|*i* = 1, 2, …, *n*}. Each student *a*_*i*_ is asked to rate how positively the other students influence him/her during group tasks. This recommendation is given on a scale from 0 to 100, where 0 (resp. 100) indicates no positive impact (resp. a very strong positive impact). The influence of student *a*_*j*_ on student *a*_*i*_ is represented as $x_{j}^{i}$. The evaluations from all students can be organized into an evaluation matrix, expressed as


$$\begin{array}{l} M=\left[\begin{array}{cccc} x_{1}^{1} & x_{1}^{2} & \ldots & x_{1}^{n}\\ x_{2}^{1} & x_{2}^{2} & \ldots & x_{2}^{n}\\ \vdots & \vdots & \ddots & \vdots \\ x_{n}^{1} & x_{n}^{2} & \ldots & x_{n}^{n} \end{array}\right] \end{array}$$

In the evaluation matrix *M*, $x_{i}^{i}$ (*i* = 1, 2, …, *n*) are set to 0.

Once the evaluation matrix has been established, following the procedure outlined in Section 2.1, the DEMATEL method is used to compute the centrality and causality indices (Ni et al., 2025). For student *a*_*i*_, the centrality index *u*_*i*_ indicates the total influence exerted by *a*_*i*_ as well as the influence received by *a*_*i*_. A high centrality index *u*_*i*_ suggests that student *a*_*i*_ is actively engaged in group tasks, reflecting their level of participation. The causality index λ_*i*_ signifies the positive impact of student *a*_*i*_ on other students. A large causality index λ_*i*_ implies that student *a*_*i*_ offers constructive feedback to others in group tasks, which highlights the participation quality of student *a*_*i*_.

When a student has high centrality and causality indices, he/she actively engages in group tasks, leading to a high score. Conversely, if the centrality and causality indices of a student are low, he/she may be free-riding and not participating actively in the group work, which results in a low score.

In the above discussion, the first requirement is adapted to the reinforcement feature of the uninorm aggregation operator, and the second requirement is adapted to the weakening feature. Since the features of reinforcement and weakening of the uninorm aggregation operator satisfy the two aforementioned requirements, the uninorm aggregation operator is used to aggregate the centrality and causality indices. The participation index Ω_*i*_ for *a*_*i*_ is determined by


(2) $${\text{Ω}}_{i}=\{\begin{array}{ll} \bar{{\text{λ}}_{i}}\times \bar{\mu_{i}}÷g & ,\;if\;0\leq \;\overset{-}{{\text{λ}}_{i}},\bar{\mu_{i}}\leq g\\ \left(\bar{{\text{λ}}_{i}}+\bar{\mu_{i}}-\overset{-}{{\text{λ}}_{i}}\times \bar{\mu_{i}}-g)÷(1-g\right) & ,\;if\;g\leq \;\overset{-}{{\text{λ}}_{i}},\bar{\mu_{i}}\leq 1\\ (\bar{{\text{λ}}_{i}}+\bar{\mu_{i}})÷2 & ,\;else \end{array}$$

where $\bar{{\text{λ}}_{i}}$ and $\bar{\mu_{i}}$ are the normalized centrality and causality indices, calculated by


(3) $$\bar{{\text{λ}}_{i}}\;=\{\begin{array}{ll} 1 & ,\;if\;{\text{Δ}}_{\text{λ}}=0\\ ({\text{λ}}_{i}-\min_{i_{1}=1,2,\ldots ,n}\{{\text{λ}}_{i_{1}}\})÷{\text{Δ}}_{\text{λ}} & ,\;else \end{array}$$

(4) $$\bar{\mu_{i}}=\{\begin{array}{ll} 1 & ,\;if\;{\text{Δ}}_{\mu }=0\\ (\mu_{i}-\min_{i_{1}=1,2,\ldots ,n}\{\mu_{i_{1}}\})÷{\text{Δ}}_{\mu } & ,\;else \end{array}$$

where ${\text{Δ}}_{\text{λ}}=\underset{i_{1}=1,2,\ldots ,n}{\max}\left\{{\text{λ}}_{i_{1}}\right\}-\underset{i_{2}=1,2,\ldots ,n}{\min}\left\{{\text{λ}}_{i_{2}}\right\}$ and ${\text{Δ}}_{\mu }=\underset{i_{1}=1,2,\ldots ,n}{\max}\left\{\mu_{i_{1}}\right\}-\underset{i_{2}=1,2,\ldots ,n}{\min}\left\{\mu_{i_{2}}\right\}$. If a student has a small composite index, then the likelihood of the student free-riding is high.

When the neutral element *g* is large, the weakening area is large (referring to Figure 1). In such a situation, students must have large centrality and causality indices (i.e., high level and quality of engagement) to obtain a large participation index. In such a context, the neutral element *g* can reflect the rigor degree of the assessment of group tasks.

### 3.2 Fair assessment index

#### 3.2.1 The unfairness index

When the differences between the participation indices of all students in a group are small, the activity level of student engagement in group tasks is about the same, which means the unfairness caused by free-riding is low. In this paper, the standard deviation of the participation indices of all students is used to represent the differences between the participation indices. The following is the definition of the unfairness index:


(5) $$F=\sqrt{\frac{\sum_{i}({\text{Ω}}_{i}-\frac{\sum_{i_{1}}{\text{Ω}}_{i_{1}}}{n}{)}^{2}}{n}}\;.$$

When the unfairness index *F* is small, the number of free-riding students in the group is few, and the individual marks of students are fair.

#### 3.2.2 The discounted score

Let δ be the score of the group given by the teacher. If the participation index Ω_*i*_ of student *a*_*i*_ is the largest participation index in the group, i.e., ${\text{Ω}}_{i}=\underset{i_{1}=1,2,\ldots ,n}{\max}\left\{{\text{Ω}}_{i_{1}}\right\}$, student *a*_*i*_ has the best comprehensive performance in group tasks, so the score of *a*_*i*_, denoted by ${\bar{s}}_{i}$, is the full score δ. If Ω_*i*_ is far away from the largest participation index, student *a*_*i*_ does not actively participate in group tasks, and there is a high probability of free-riding. To ensure fairness, the score of *a*_*i*_ should have a discount. Let ψ ∈ [0, 1] be the largest discount rate determined by teachers. The discount parameter of student *a*_*i*_ is defined as


(6) $$\begin{array}{l} \xi_{i}=\left(1-\psi \right)\times {\text{Ω}}_{i}+\psi \end{array}$$

The discounted score of *a*_*i*_ is calculated by


(7) $$\begin{array}{l} {\bar{s}}_{i}=\xi_{i}\times \delta \end{array}$$

** Example 2**. ***(Example for using the fair assessment framework to uncover the free-riding behavior)***. Suppose there are 3 students in a group. The second student has free-riding behavior, meaning he/she is not actively participating in the group task. Given that the second student is free-riding, the first and third students give low evaluations to the second, resulting in small values in the second row of *M*. The second student gives the same value of 85 to the first and third students. The evaluation matrix is given as


$$\begin{array}{l} M=\left[\begin{array}{ccc} 0 & 85 & 90\\ 60 & 0 & 65\\ 85 & 85 & 0 \end{array}\right] \end{array}$$

Referring to Step 2 in Section 2.1, the normalized evaluation matrix is calculated by *M*/*S* where *S* = max{max{145, 170, 155}, max{175, 125, 170}} = 175, which is shown as


$$\begin{array}{l} \bar{M}=\left[\begin{array}{ccc} 0 & 0.4857 & 0.5143\\ 0.3429 & 0 & 0.3714\\ 0.4857 & 0.4857 & 0 \end{array}\right] \end{array}$$

Referring to Step 3 in Section 2.1, the direct total influence matrix is calculated as


$$\begin{array}{l} T=\left[\begin{array}{ccc} 2.5635 & 3.198 & 3.0205\\ 2.2751 & 2.2618 & 2.3816\\ 2.8359 & 3.1376 & 2.6239 \end{array}\right] \end{array}$$

For student *a*_1_, the centrality index μ_1_ (resp. causality index λ_1_) is calculated as μ_1_ = (2.5635 + 3.198 + 3.0205) + (2.5635 + 2.2751 + 2.8359) = 16.4565 (resp. λ_1_ = (2.5635 + 3.198 + 3.0205)−(2.5635 + 2.2751 + 2.8359) = 1.1073). For student *a*_2_, the centrality index μ_2_ (resp. causality index λ_2_) is calculated as μ_2_ = (2.2751 + 2.2618 + 2.3816) + (3.198 + 2.2618 + 3.1376) = 15.516 (resp. λ_2_ = (2.2751 + 2.2618 + 2.3816)−(3.198 + 2.2618 + 3.1376) = −1.6788). For student *a*_3_, the centrality index μ_3_ (resp. causality index λ_3_) is calculated as μ_3_ = (2.8359 + 3.1376 + 2.6239) + (3.0205 + 2.3816 + 2.2639) = 16.6234 (resp. λ_3_ = (2.8359 + 3.1376 + 2.6239)−(3.0205 + 2.3816 + 2.2639) = 0.5714).

Referring to Equations 3, 4, the normalized centrality and causality indices for the three students are calculated as ${\bar{\mu }}_{1}=0.8494$, ${\bar{\text{λ}}}_{1}=1$, ${\bar{\mu }}_{2}=0$, ${\bar{\text{λ}}}_{2}=0$, ${\bar{\mu }}_{3}=1$, and ${\bar{\text{λ}}}_{3}=0.8076$. Let the neutral element *g* be 0.5. According to Equation 2, the participation indices of the three students are Ω_1_ = 1, Ω_2_ = 0, and Ω_3_ = 1. Here, the free-riding behavior of the second student is revealed by Ω_2_ = 0.

Referring to Equation 5, the unfairness index for the group is about 0.47. The teacher gives a score of 90 to the group. The largest discount rate is set to 0.6, which means that every student in the group will receive a minimum score of 90 × 0.6 = 54. Referring to Equation 7, the discounted scores of *a*_1_, *a*_2_, and *a*_3_ are 90, 54, and 90, respectively.

## 4 Case study

This section applies the previously described methodology to a real course (*Optimization Theory and Method*) setting to demonstrate its feasibility in identifying and addressing free-riding behavior in flipped classrooms. Specifically, Section 4.1 introduces the background of the case study and presents the collected data. In Section 4.2, we employ the uninorm DEMATEL method to evaluate student performance. Section 4.3 analyzes the influence of the neutral element on the unfairness index and the discounted scores.

### 4.1 Background and data collection

#### 4.1.1 Implementation of the flipped classroom

To systematically investigate individual engagement and collaborative learning outcomes in group tasks, this study adopted *Optimization Theory and Methods* as the case study to demonstrate the feasibility of the proposed fair assessment framework.

The *Optimization Theory and Methods* involves 57 students and one teacher, which serves as a core theoretical course and a designated pilot for the institution's flipped classroom initiative. Initially, students were allowed to form teams freely. A requirement was that there should be at least two students in a team. The results of the grouping were shown in Table 1. Following team formation, the instructor implemented a group task framework requiring each team to:

**Table 1:** Grouping information and group scores.

**Group number**	**Number of students**	**Students**	**Group score**
1	2	*a*_*i*_, *i* = 1, 2	95
2	3	*a*_*i*_, *i* = 3, 4, 5	90
3	4	*a*_*i*_, *i* = 6, 7, 8, 9	85
4	3	*a*_*i*_, *i* = 10, 11, 12	80
5	3	*a*_*i*_, *i* = 13, 14, 15	85
6	4	*a*_*i*_, *i* = 16, 17, 18, 19	88
7	4	*a*_*i*_, *i* = 20, 21, 22, 23	83
8	4	*a*_*i*_, *i* = 24, 25, 26, 27	80
9	7	*a*_*i*_, *i* = 28, 29, 30, 31, 32, 33, 34	75
10	6	*a*_*i*_, *i* = 35, 36, 37, 38, 39, 40	85
11	6	*a*_*i*_, *i* = 41, 42, 43, 44, 45, 46	78
12	4	*a*_*i*_, *i* = 47, 48, 49, 50	70
13	2	*a*_*i*_, *i* = 51, 52	85
14	5	*a*_*i*_, *i* = 53, 54, 55, 56, 57	87

(1) Selecting a seminal paper from the optimization theory and methods literature.

(2) Analyzing the chosen paper through at least five lenses: (a) Research rationale and theoretical foundations; (b) Contextualization of problem background; (c) Implementation and validation of methodological frameworks; (d) Interpretation and presentation of results/conclusions; and (e) Identification of methodological limitations.

(3) Proposing improvement strategies after analyzing the chosen paper.

During pre-class preparation, each group collaboratively selected a paper to present, considering factors such as alignment with course objectives, thematic relevance, and academic value. Subsequently, specific tasks and responsibilities were assigned to individual group members, and presentation materials (e.g., PowerPoint slides) were prepared accordingly. During in-class learning activities, each group delivered a presentation of 15 to 20 min. The lead speaker provided the primary explanation, while other members offered supplementary insights and responded to questions when necessary. Both the teacher and classmates were encouraged to engage in discussion and pose questions. In the post-class consolidation phase, groups revised their presentations based on feedback received during class and submitted a final learning report.

#### 4.1.2 Data collection

In the above instructional design, teachers often faced difficulties in directly engaging with each group's internal activities, making it challenging to identify free-riding students. To promote fairness in evaluating student performance, each student was required to assess the extent to which other group members positively influenced their learning. Evaluations were provided on a scale from 0 to 100, where 0 indicated no positive impact and 100 indicated a strong positive impact.

Intra-group students' evaluations were collected using an online platform (named as *Wenjuanxing*) to ensure anonymity and confidentiality. The questionnaire was distributed and retrieved by members of the research team. The detailed evaluations for each group are presented in Table 2.

**Table 2:** Intra-group evaluations within each group.

**Group number**	**Evaluation matrix**	**Group number**	**Evaluation matrix**
1	$M_{1}=\left[\begin{array}{cc} 0 & 99\\ 90 & 0 \end{array}\right]$	2	$M_{2}=\left[\begin{array}{ccc} 0 & 85 & 80\\ 90 & 0 & 94\\ 99 & 99 & 0 \end{array}\right]$
3	$M_{3}=\left[\begin{array}{cccc} 0 & 98 & 98 & 98\\ 98 & 0 & 98 & 98\\ 98 & 98 & 0 & 98\\ 99 & 98 & 98 & 0 \end{array}\right]$	4	$M_{4}=\left[\begin{array}{ccc} 0 & 100 & 98\\ 95 & 0 & 95\\ 80 & 95 & 0 \end{array}\right]$
5	$M_{5}=\left[\begin{array}{ccc} 0 & 93 & 91\\ 76 & 0 & 85\\ 95 & 85 & 0 \end{array}\right]$	6	$M_{6}=\left[\begin{array}{cccc} 0 & 100 & 100 & 98\\ 98 & 99 & 0 & 97\\ 98 & 100 & 0 & 98\\ 92 & 100 & 92 & 0 \end{array}\right]$
7	$M_{7}=\left[\begin{array}{cccc} 0 & 92 & 92 & 94\\ 89 & 87 & 0 & 88\\ 91 & 90 & 0 & 90\\ 86 & 88 & 86 & 0 \end{array}\right]$	8	$M_{8}=\left[\begin{array}{cccc} 0 & 95 & 95 & 95\\ 90 & 90 & 0 & 90\\ 78 & 80 & 0 & 82\\ 95 & 95 & 95 & 0 \end{array}\right]$
9	$M_{9}=\left[\begin{array}{ccccccc} 0 & 98 & 98 & 98 & 98 & 98 & 98\\ 95 & 0 & 95 & 95 & 95 & 95 & 95\\ 99 & 99 & 0 & 99 & 99 & 99 & 99\\ 94 & 92 & 93 & 0 & 92 & 92 & 92\\ 90 & 90 & 90 & 90 & 0 & 90 & 90\\ 94 & 94 & 94 & 94 & 94 & 0 & 94\\ 92 & 92 & 92 & 92 & 92 & 92 & 0 \end{array}\right]$	10	$M_{10}=\left[\begin{array}{cccccc} 0 & 99 & 99 & 99 & 99 & 99\\ 95 & 0 & 94 & 94 & 95 & 95\\ 99 & 99 & 0 & 99 & 99 & 99\\ 93 & 94 & 94 & 0 & 100 & 100\\ 92 & 92 & 94 & 94 & 0 & 100\\ 98 & 98 & 98 & 98 & 100 & 0 \end{array}\right]$
11	$M_{11}=\left[\begin{array}{cccccc} 0 & 91 & 96 & 91 & 90 & 90\\ 92 & 0 & 96 & 91 & 90 & 90\\ 96 & 94 & 0 & 96 & 94 & 95\\ 99 & 99 & 99 & 0 & 99 & 99\\ 90 & 90 & 90 & 90 & 0 & 95\\ 90 & 90 & 80 & 90 & 95 & 0 \end{array}\right]$	12	$M_{12}=\left[\begin{array}{cccc} 0 & 98 & 98 & 100\\ 98 & 0 & 98 & 98\\ 98 & 98 & 0 & 98\\ 90 & 91 & 91 & 0 \end{array}\right]$
13	$M_{13}=\left[\begin{array}{cc} 0 & 95\\ 99 & 0 \end{array}\right]$	14	$M_{14}=\left[\begin{array}{ccccc} 0 & 100 & 100 & 100 & 100\\ 100 & 0 & 100 & 100 & 60\\ 100 & 100 & 0 & 100 & 100\\ 100 & 100 & 100 & 0 & 100\\ 100 & 98 & 98 & 98 & 0 \end{array}\right]$

#### 4.1.3 Ethical statements

Ethical review and approval were not required for the study on human participants in accordance with the local legislation and institutional requirements. Written informed consent to participate in this study was provided by the participants.

Before collecting data, students were presented with an informed consent form embedded within the online platform, granting permission for their data to be used for research purposes. Students who felt uncomfortable were allowed to withdraw from the survey or abstain from providing scores. During data collection, the research team ensured the protection of students' privacy, confidentiality, anonymity, and non-traceability.

### 4.2 Case study solving

In this section, we calculate the unfairness index of each group and generate the discount parameter for every student through the uninorm DEMATEL method. The neutral element *g* is set to 0.5. The teacher gives the largest discount rate ψ of 0.6.

We take the first group as an example to show how the unfairness index and the discounted scores are calculated. For the first group, referring to Step 2 in Section 2.1, the normalized evaluation matrix is calculated by ${\bar{M}}_{1}=M_{1}/S_{1}$ where *S*_1_ = max{max{99, 90}, max{99, 90}} = 99, shown as


$$\begin{array}{l} {\bar{M}}_{1}=\left[\begin{array}{cc} 0 & 1\\ 0.9091 & 0 \end{array}\right]\;. \end{array}$$

Referring to Step 3 in Section 2.1, the total influence matrix for the first group is calculated as


$$\begin{array}{l} T_{1}=\left[\begin{array}{cc} 10 & 11\\ 10 & 10 \end{array}\right]\;. \end{array}$$

For student *a*_1_, the centrality index μ_1_ (resp. the causality index λ_1_) is calculated as μ_1_ = (10 + 11) + (10 + 10) = 41 (resp. λ_1_ = (10 + 11)−(10 + 10) = 1). For student *a*_2_, the centrality index μ_2_ (resp. the causality index λ_2_) is μ_2_ = 41 (resp. λ_2_ = −1).

Referring to Equations 3, 4, the normalized centrality and causality indices of *a*_1_ and *a*_2_ are calculated as ${\bar{\mu }}_{1}=1$, ${\bar{\text{λ}}}_{1}=1$, ${\bar{\mu }}_{2}=1$, and ${\bar{\text{λ}}}_{2}=0$. According to Equation 2, the participation indices of *a*_1_ and *a*_2_ are 1 and 0.5, respectively. Referring to Equation 7, the discounted scores of *a*_1_ and *a*_2_ are calculated as 95 and 76. The unfairness index of the first group is calculated as $F_{1}=\sqrt{\left[{\left(1-0.75\right)}^{2}+{\left(0.5-0.75\right)}^{2}\right]÷2}=0.25$.

**Figure 3:**
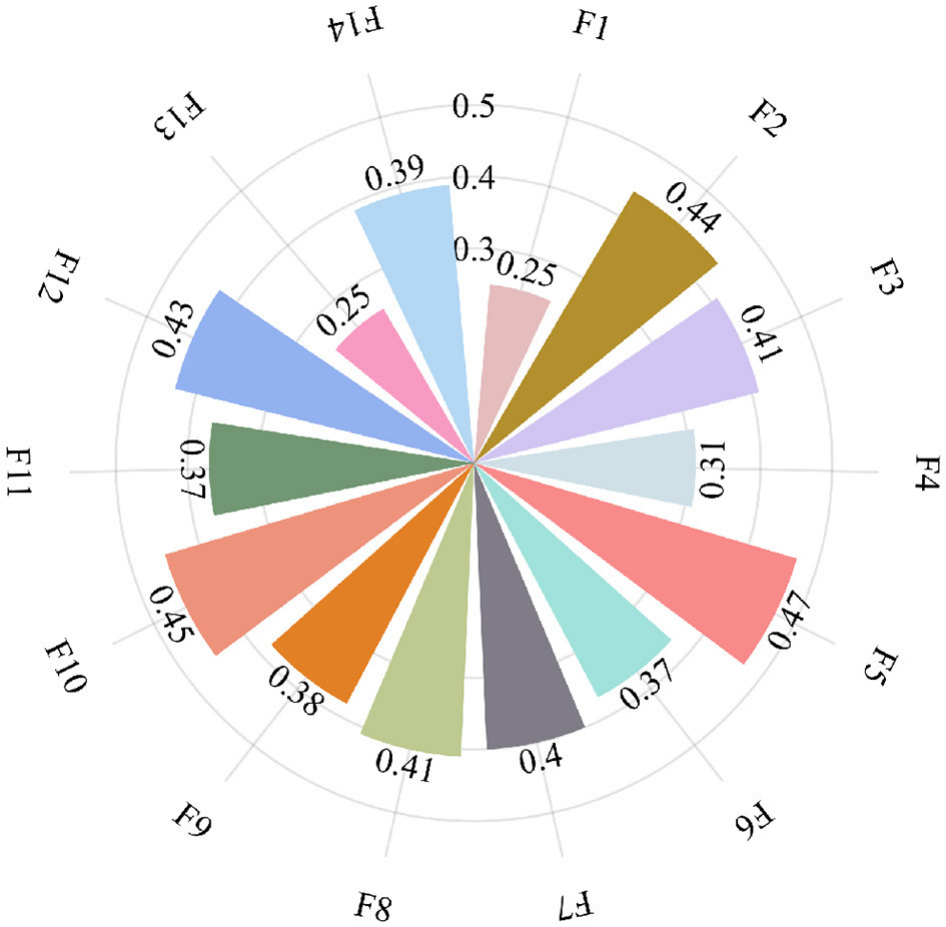
The unfairness index of 14 groups.

The unfairness index for the other groups is calculated in the same way, and the final scores for each of the 14 groups are *F*_1_ = 0.25, *F*_2_ = 0.44, *F*_3_ = 0.41, *F*_4_ = 0.31, *F*_5_ = 0.47, *F*_6_ = 0.37, *F*_7_ = 0.4, *F*_8_ = 0.41, *F*_9_ = 0.38, *F*_10_ = 0.45, *F*_11_ = 0.37, *F*_12_ = 0.43, *F*_13_ = 0.25, and *F*_14_ = 0.39, as shown in Figure 3.

**Figure 4:**
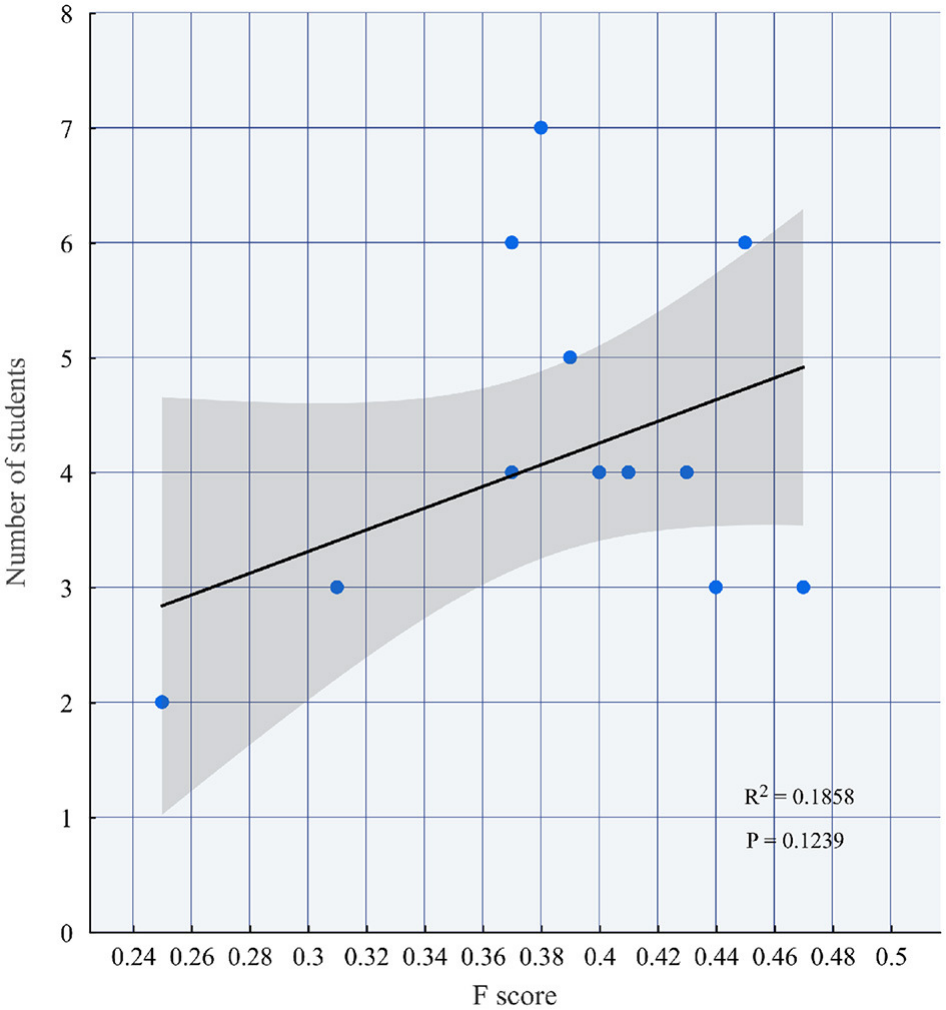
The relationship between the number of students and the *F* score.

Further analysis reveals that the number of students does not exhibit a direct correlation with unfairness indices, as statistically confirmed by the nonsignificant *p*-value (*p* = 0.1239) in Figure 4. It can be known that when the number of students in the group is equal to 2, the unfairness index (i.e., the *F* score) is low (< 0.26). When the number of students in the group is equal to 3, the unfairness index varies from a low unfairness index of about 0.3 to a high unfairness index of about 0.5. For the medium-to-large-sized groups (4 − 7 members), the unfairness index is at least a medium level (>0.36).

The discount rate of the 14 groups revealed that there were significant differences in the fairness levels of the groups (The value range: 0.25 − 0.47), reflecting the dynamic characteristics of the recognition of contribution distribution in group collaboration. Among them, the smallest 2-member group (Group 1 and Group 13) had the best fairness (*F*_1_ = *F*_13_ = 0.25), indicating that small-scale collaboration is more likely to achieve transparent division of labor and consensus; while Group 5, consisting of 3 members, had the worst fairness (*F*_5_ = 0.47), probably due to the large divergence in members' evaluations or the existence of free-riding behaviors. Further analysis showed that group size did not show a simple linear relationship with fairness: the unfairness indices (i.e., *F*) of medium-to-large-sized groups (4–7 members) were mostly concentrated in the range of 0.37–0.45, indicating that although more members increase the complexity of collaboration, moderate fairness can still be maintained through effective management (e.g., *F*_9_ = 0.38 for the group of 7 members).

**Figure 5:**
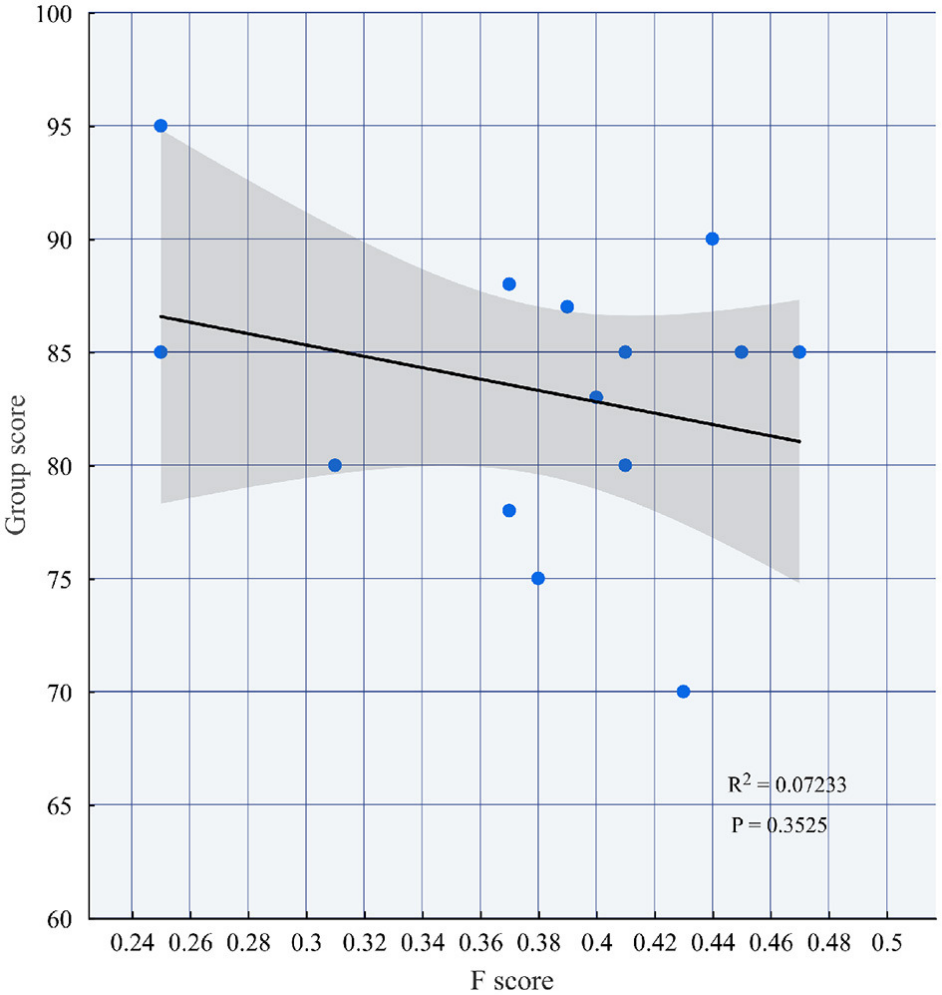
The relationship between group scores and *F* scores.

The analysis reveals that group scores do not exhibit direct correlation with unfairness indices, as statistically confirmed by the nonsignificant *p*-value (*p* = 0.3525) in Figure 5. More specifically, high Group 14 (Score = 87) still has moderate inequity (*F*_14_ = 0.39), while the high unfairness index of low Group 12 (Score = 70) (*F*_12_ = 0.43) suggests that its underperformance may be superimposed on the imbalance in the distribution of contributions. The results of this paper emphasize that the inequity index can provide a quantitative basis for identifying collaboration problems, and teachers need to focus on groups with *F*>0.4 (e.g., Groups 2, 5, 10, and 12) to optimize the team division of labor mechanism and dynamic monitoring strategies, thus enhancing the fairness of course assessment and the achievement of educational goals.

The discounted scores presented in Table 3 demonstrate the practical implications of integrating the unfairness index into academic assessments. Significant score variations within high-unfairness-index groups (e.g., Group 5, *F*_5_ = 0.47) highlight pronounced disparities in perceived contributions, as evidenced by contrasting outcomes such as Student *a*_14_ (Score = 51) vs. *a*_13_ and *a*_15_ (Score = 85), suggesting potential free-riding or evaluation conflicts. Conversely, low-unfairness-index groups (e.g., Group 1, *F*_1_ = 0.25) exhibit minimal score adjustments (e.g., *a*_1_ = 95, *a*_2_ = 76), reinforcing that smaller team sizes enhance transparency and consensus. Notably, even high-performing groups (e.g., Group 14, score = 87) show moderate unfairness (*F*_14_ = 0.39), with substantial discounts for some students (e.g., *a*_54_ = 52.2 versus *a*_55_ = 85.608), underscoring that academic excellence does not inherently ensure equitable contribution distribution. These findings validate the unfairness index as a robust tool for identifying collaboration inefficiencies, particularly in groups with *F*>0.4 (e.g., Groups 5 and 12), where extreme discounts (e.g., *a*_50_ = 42) signal urgent needs for pedagogical interventions. This approach equips educators with actionable insights to refine team dynamics and align assessment outcomes with educational equity goals.

**Table 3:** Discounted scores of all students.

**Student**	**Discountedscore**	**Student**	**Discountedscore**	**Student**	**Discountedscore**	**Student**	**Discountedscore**	**Student**	**Discountedscore**
*a*_1_	95	*a*_13_	85	*a*_25_	72	*a*_37_	83.64	*a*_49_	69.72
*a*_2_	76	*a*_14_	51	*a*_26_	48	*a*_38_	61.54	*a*_50_	42
*a*_3_	54	*a*_15_	85	*a*_27_	80	*a*_39_	51	*a*_51_	68
*a*_4_	83.52	*a*_16_	88	*a*_28_	74.4	*a*_40_	85	*a*_52_	85
*a*_5_	90	*a*_17_	73.216	*a*_29_	63.3	*a*_41_	51.48	*a*_53_	87
*a*_6_	68	*a*_18_	80.608	*a*_30_	75	*a*_42_	51.48	*a*_54_	52.2
*a*_7_	51	*a*_19_	52	*a*_31_	49.8	*a*_43_	68.016	*a*_55_	85.608
*a*_8_	51	*a*_20_	83	*a*_32_	45	*a*_44_	78	*a*_56_	85.608
*a*_9_	85	*a*_21_	52.788	*a*_33_	57	*a*_45_	49.608	*a*_57_	69.6
*a*_10_	68.8	*a*_22_	68.06	*a*_34_	48	*a*_46_	46.8		
*a*_11_	69.12	*a*_23_	49	*a*_35_	85.1	*a*_47_	70		
*a*_12_	48	*a*_24_	80	*a*_36_	51	*a*_48_	69.72		

### 4.3 Sensitivity analysis

**Figure 6:**
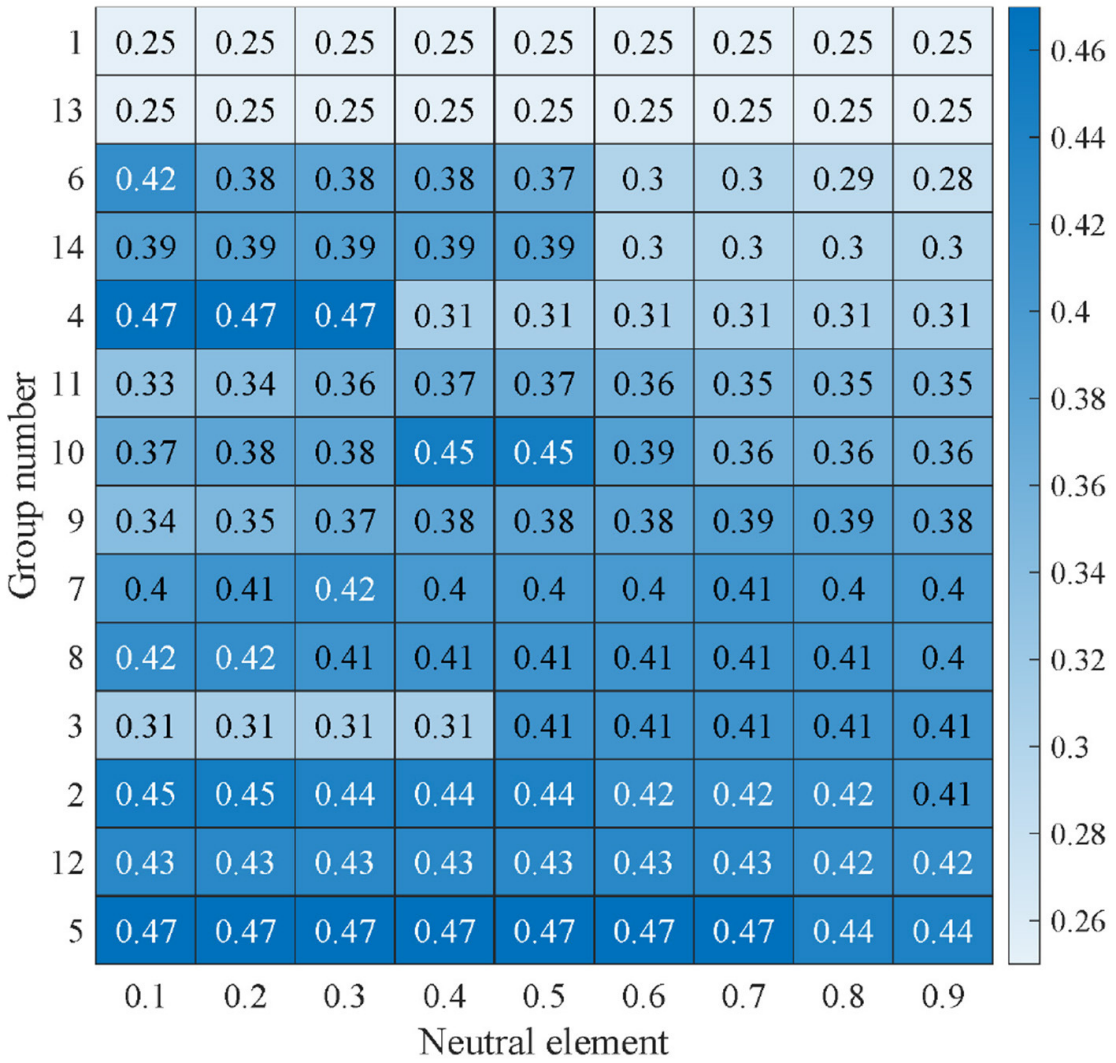
The unfairness indices with different neutral elements.

This section performs the sensitivity analysis to discover the influence of the neutral element on unfairness indices and discounted scores. The neutral element is set to 0.1, 0.2, 0.3, 0.4, 0.5, 0.6, 0.7, 0.8, and 0.9. The unfairness indices with different neutral elements are shown in Figure 6.

The means and standard variances of the unfairness indices of the 14 group are shown in Table 4. The sensitivity analysis results show that the variation of the neutral element (*g*) has a limited overall impact on the unfairness index, which verifies the advantages of the uninorm DEMATEL method in terms of parameter robustness. The mean of unfairness indices fluctuations of the 14 groups under different neutral elements are narrower (0.25–0.4633), and the standard deviation is generally lower (e.g., the standard deviation of Groups 1 and 13 is 0, and the others are mostly lower than 0.05). This indicates that although the adjustment of the neutral element slightly affects the quantitative results of fairness, it does not trigger a significant systematic bias, suggesting that the method is insensitive to parameter selection.

**Table 4:** Means and standard variances of the unfairness indices.

**Group**	**Mean**	**Std**.	**Group**	**Mean**	**Std**.
1	0.25	0	8	0.4111	0.0057
2	0.4322	0.014	9	0.3733	0.0163
3	0.3656	0.0497	10	0.3889	0.0341
4	0.3633	0.0754	11	0.3533	0.0125
5	0.4633	0.0125	12	0.4278	0.0042
6	0.3444	0.0486	13	0.25	0
7	0.4044	0.0068	14	0.35	0.0447

**Figure 7:**
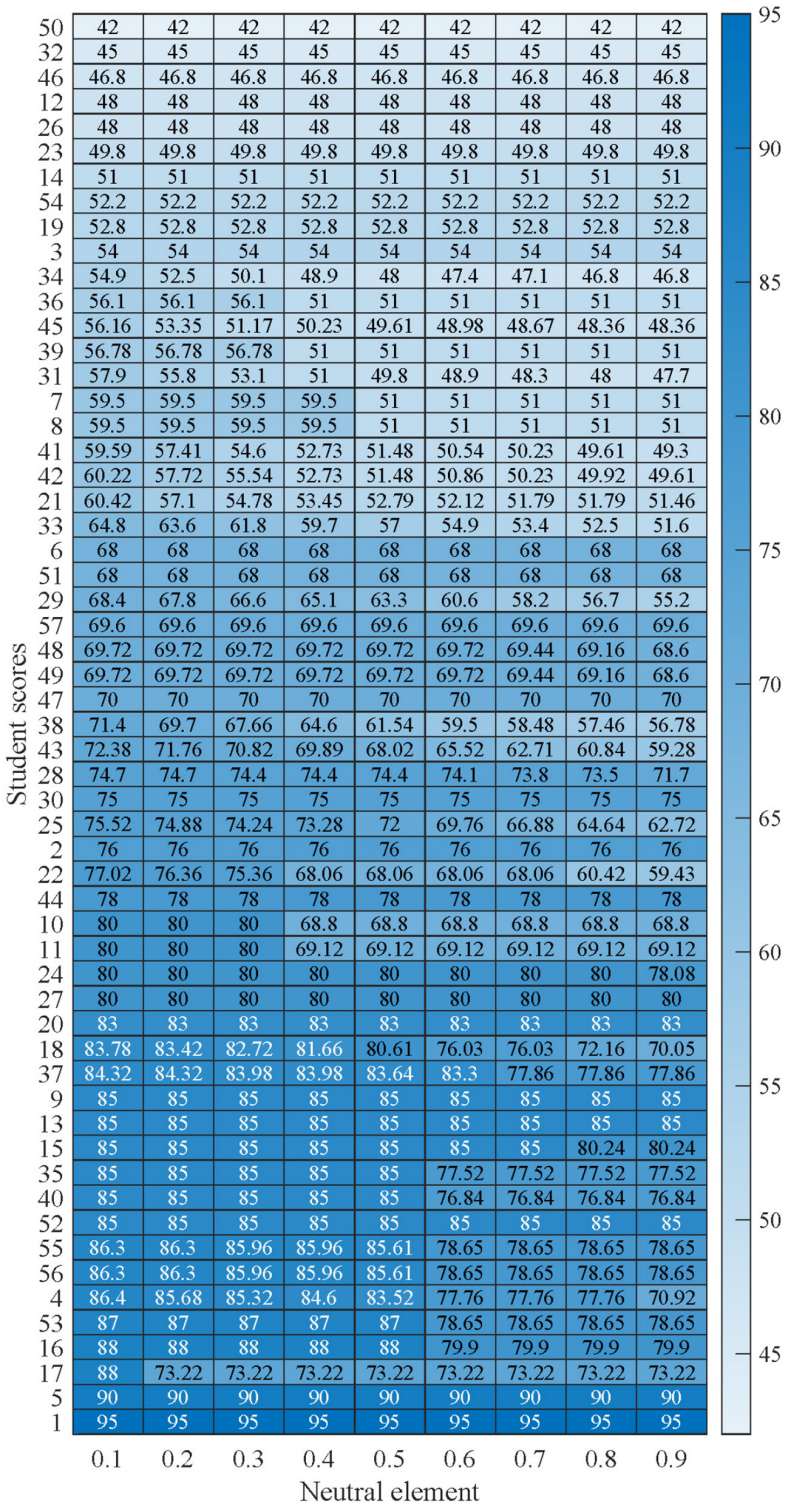
The discounted scores with different neutral elements.

**Figure 8:**
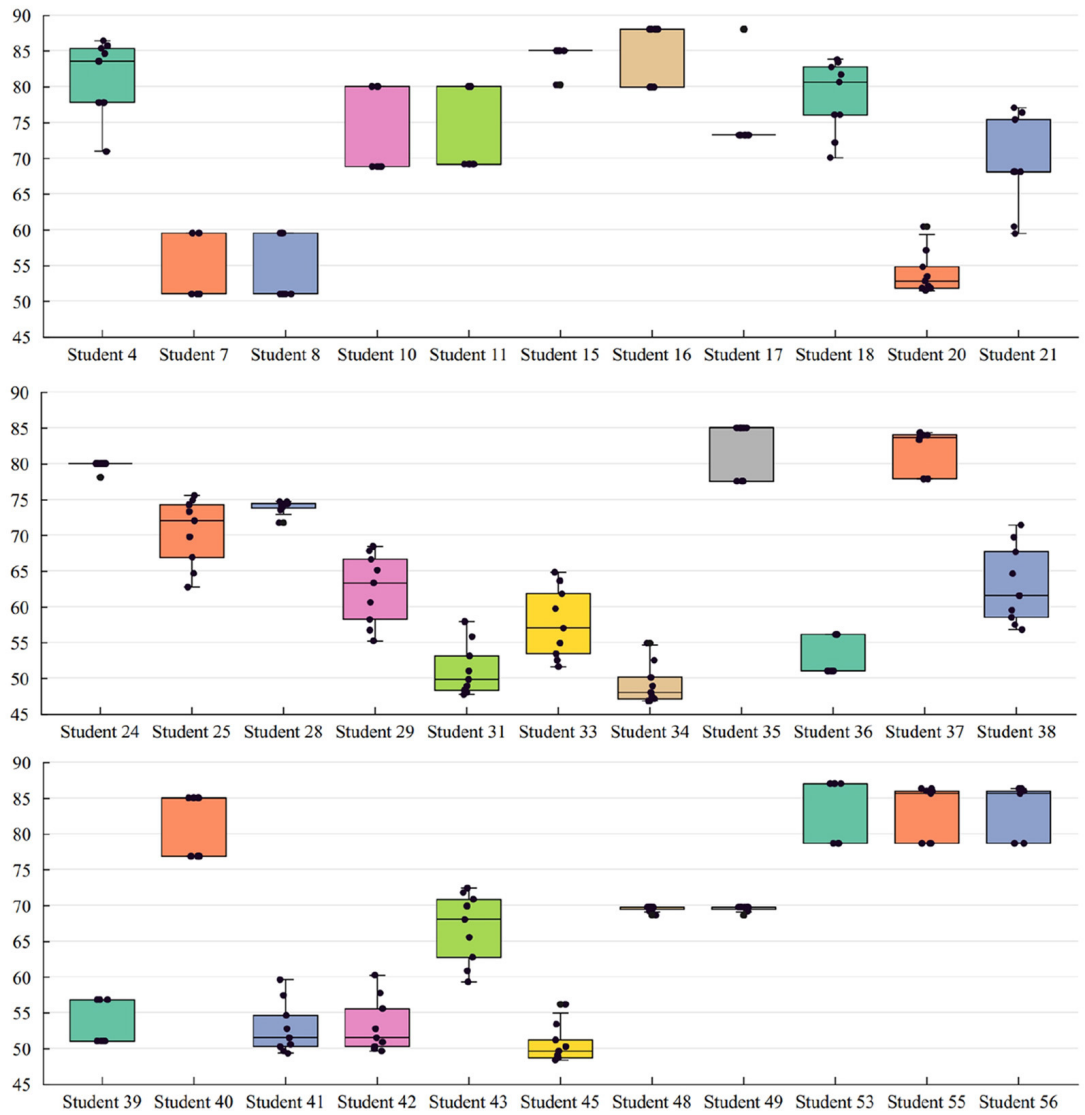
Distribution of students' scores with different neutral elements.

Then, the discounted scores of all students with different neutral elements are calculated, which are shown in Figure 7. Of all the students, those whose scores changed with the neutral element are shown in Figure 8. The means and standard variances of the discounted scores of the 57 students are given in Table 5, and the following findings can be drawn:

**Table 5:** Means and standard variances of the discounted scores.

**Student**	**(Mean,Std.)**	**Student**	**(Mean,Std.)**	**Student**	**(Mean,Std.)**	**Student**	**(Mean,Std.)**	**Student**	**(Mean,Std.)**
*a*_1_	(95,0)	*a*_13_	(48,0)	*a*_25_	(70.44,4.43)	*a*_37_	(81.9,2.87)	*a*_49_	(69.5,0.37)
*a*_2_	(76,0)	*a*_14_	(85,0)	*a*_26_	(48,0)	*a*_38_	(63.01,5.21)	*a*_50_	(42,0)
*a*_3_	(54,0)	*a*_15_	(51,1.98)	*a*_27_	(80,0)	*a*_39_	(52.93,2.72)	*a*_51_	(68,0)
*a*_4_	(81,4.97)	*a*_16_	(83.94,4.02)	*a*_28_	(73.97,0.88)	*a*_40_	(81.37,4.05)	*a*_52_	(85,0)
*a*_5_	(90,0)	*a*_17_	(84.4,4.65)	*a*_29_	(62.43,4.67)	*a*_41_	(52.83,3.44)	*a*_53_	(83.29,4.15)
*a*_6_	(68,0)	*a*_18_	(74.86,4.81)	*a*_30_	(75,0)	*a*_42_	(53.14,3.59)	*a*_54_	(52.2,0)
*a*_7_	(54.78,4.22)	*a*_19_	(78.5,0)	*a*_31_	(51.17,3.46)	*a*_43_	(66.8,4.64)	*a*_55_	(82.75,3.67)
*a*_8_	(54.78,4.22)	*a*_20_	(52.8,0)	*a*_32_	(45,0)	*a*_44_	(78,0)	*a*_56_	(82.75,3.67)
*a*_9_	(51,0)	*a*_21_	(53.97,2.85)	*a*_33_	(57.7,4.68)	*a*_45_	(50.54,2.5)	*a*_57_	(69.6,0)
*a*_10_	(85,5.28)	*a*_22_	(68.98,6.03)	*a*_34_	(49.17,2.68)	*a*_46_	(46.8,0)		
*a*_11_	(72.53,5.13)	*a*_23_	(49.8,0)	*a*_35_	(81.68,3.72)	*a*_47_	(70,0)		
*a*_12_	(72.75,0)	*a*_24_	(79.79,0.6)	*a*_36_	(52.7,2.4)	*a*_48_	(69.5,0.37)		

(1) The proposed model is robust to changes in the neutral element (*g*). Many of the students' discounted scores in the table remain stable under different *g*, and both the mean and standard deviation show low volatility. For example, the discounted scores of students *a*_1_ and *a*_2_ have mean values of 95 and 76, respectively, and a standard deviation of 0, indicating that their discounted scores are not affected at all regardless of changes in *g*. Also, the results from 22 other students further show that the model is stable.

(2) The standard deviations are generally low, indicating that the model outputs are not sensitive to the choice of parameters. Most of the students have standard deviations lower than 5. For example, Student *a*_37_ has a discounted score mean of 81.9 and a standard deviation of 2.87, indicating that the range of fluctuation of his discounted score across different *g* is small. The mean value of the discounted scores of Student *a*_49_ is 69.5, and the standard deviation is only 0.37, which further indicates the stability of the model.

(3) Even if there are individual students whose discounted scores fluctuate greatly, their impact is still within a reasonable range. For example, the discounted score of student *a*_25_ has a mean value of 70.44 and a standard deviation of 4.43. Although the fluctuation is a bit large, the mean value is still within a reasonable range, which shows that the model can effectively deal with the effects of parameter changes.

In summary, the sensitivity analysis proves the robustness of the uninorm DEMATEL method in parameter selection, which provides a reliable guarantee for the practical application of the model.

## 5 Discussion

This paper proposes a fair assessment framework to evaluate individual engagement and collaborative learning outcomes in group tasks within university flipped classrooms. The framework employs an unfairness index and discounted scores to identify potential free-riding behaviors and quantify the level of fairness within each group. Below is a detailed discussion of the findings and their implications:

(1) Free-riding behavior exists in university flipped classrooms, and quantifying unfairness enables more targeted pedagogical interventions. A key contribution of this study is the development of a quantifiable unfairness index (*F*), which serves as a precise diagnostic tool for evaluating group collaboration (Benning, 2024). The case study demonstrates significant variation in unfairness indices across groups (ranging from 0.25 to 0.47), consistent with previous research that highlights the persistent challenge of achieving equitable contribution distribution in collaborative learning environments (Hincapie and Costa, 2024; Ion et al., 2024). The unfairness index enables instructors to detect potential free-riding behavior and prioritize high-risk groups (e.g., those with *F*>0.4), thereby ensuring that assessment outcomes are aligned with principles of fairness and educational equity.

(2) Discounted scores can enhance student motivation while promoting fairness. Unlike traditional peer review systems that primarily provide qualitative feedback or serve as secondary evaluation inputs (Cheng and Zhang, 2024), discounted scores quantitatively adjust individual grades based on relative contribution levels, thus reinforcing personal accountability in group tasks. Notably, significant score discrepancies were observed in groups with high unfairness indices. For example, in Group 5 (*F*_5_ = 0.47), Student *a*_14_ received a score of 51, while *a*_13_ and *a*_15_ each scored 85, suggesting potential free-riding behavior or intra-group evaluation conflicts. The use of discounted scores makes students aware that unequal effort will directly affect their final grades (Holm, 2025; Luo and Wang, 2025), thereby incentivizing more active participation. This mechanism is more effective in curbing free-riding than verbal reminders or peer pressure alone. Moreover, many students report concerns about fairness in group assignments—such as receiving identical grades despite unequal contributions (Salza, 2022). Discounted scores directly address this concern by ensuring that both group outcomes and individual efforts are reflected in the final assessment.

(3) Group scores do not show a direct correlation with unfairness indices. Intuitively, when the difference in the degree of participation within a group (i.e., the *F* score) is large, the ability of the group to complete group tasks is low, and therefore the score of the group is low. This is evident in Group 12, which recorded an unfairness index of *F*_12_ = 0.43 and a relatively low group score of 70. Interestingly, even high-scoring groups can exhibit moderate levels of unfairness. For instance, Group 14 achieved a score of 87 but still recorded an unfairness index of *F*_14_ = 0.39, with the lowest discounted score being *a*_54_ = 52.2. This suggests that high group performance does not necessarily imply equitable individual contributions (Lünich et al., 2024).

(4) The number of students does not exhibit a direct correlation with unfairness indices. Although detailed analysis reveals that larger groups are more likely to exhibit free-riding behavior, the observed difference did not reach statistical significance. This finding is consistent with classical social loafing theory (Karau and Williams, 1993), which posits that reduced individual accountability in larger groups promotes free-riding behaviors. In case study, when the number of students is greater than 3, the unfairness index of the group is relatively high, which indicates that there are students in the group who do not participate in the group tasks and reduces the effect of the flipped classroom. Therefore, when conducting the flipped classroom, it is recommended that the number of students in each group should not be too large.

## 6 Conclusion

This paper integrates intra-group student evaluations and teacher assessments to propose a fair assessment framework incorporating the uninorm DEMATEL method, an unfairness index, and a discounted score. Specifically, the DEMATEL method is employed to compute the centrality and causality indices of students, which reflect the level and quality of their engagement. The unfairness index, defined as the standard deviation of participation indices, provides instructors with a quantitative tool to assess individual engagement and identify free-riding behavior. The discounted score adjusts the outcomes of students who contribute less to group tasks, serving as a corrective measure to address disparities in contribution and reinforce fairness in evaluation. A case study demonstrates the practicality and effectiveness of the proposed framework, while sensitivity analysis confirms its robustness. Overall, the proposed framework promotes collaborative learning and supports the effective implementation of flipped classrooms, thereby contributing to the sustainable development of higher education.

## 7 Limitations and future research

In this paper, only one course involving 57 students (*Optimization Theory and Method*) was analyzed. Future research will aim to expand the sample size and compare the differences among general education courses, major-specific courses, and elective courses to uncover additional insights. Second, this study assumes that students provide authentic evaluation scores. However, in real-world settings, students may submit biased or dishonest evaluations in an attempt to lower the grades of their intra-group members. Detecting and mitigating such manipulative behaviors remains an important direction for future research. To achieve more comprehensive and objective evaluations, future work should also incorporate inter-group evaluations beyond the group and teacher evaluations.

## Data Availability

The original contributions presented in the study are included in the article/supplementary material, further inquiries can be directed to the corresponding author.
